# Prevalence of Musculoskeletal Disorders Among Dentists in Primary Healthcare Settings in Qatar: A Cross-Sectional Study

**DOI:** 10.7759/cureus.87257

**Published:** 2025-07-03

**Authors:** Abir Eddhaoui, Muslim Abbas Syed

**Affiliations:** 1 Department of Dentistry, Primary Health Care Corporation, Doha, QAT; 2 Clinical Research Department, Primary Health Care Corporation, Doha, QAT

**Keywords:** cross-sectional study, dentistry, ergonomics, musculoskeletal disorders, occupational health, primary healthcare, qatar, work-related injury

## Abstract

Introduction

Musculoskeletal disorders (MSDs) are prevalent among dental professionals due to the physical demands of clinical work, including awkward postures and repetitive motions. These conditions pose a risk to both practitioner well-being and service continuity. In Qatar, however, data on the extent of this issue within primary healthcare settings remains limited. This study investigates the prevalence of MSDs among dentists at the Primary Health Care Corporation (PHCC) and explores contributing occupational factors.

Methods

A cross-sectional study was conducted to assess the prevalence of MSDs among dentists working at PHCC in Qatar. Data were collected via a structured, self-administered online questionnaire disseminated through official PHCC email accounts using Microsoft Forms (Redmond, WA, USA). All dentists with active official email addresses including general dentists, specialists, and consultants were invited to participate in the study between November 1 and November 30, 2024. A follow-up reminder was sent two weeks after the initial invitation to encourage participation. The questionnaire consisted primarily of structured, closed-ended questions, with one open-ended item at the end to collect suggestions for improvement. It addressed six thematic areas: demographic information, musculoskeletal symptoms, interventions used, impact on work, ergonomic awareness and practices, and participant recommendations.

Results

Of the 42 respondents, 33 (78.6%) reported musculoskeletal symptoms within the past year. Among them, 15 (45.5%) required sick leave due to these symptoms. Twenty-one (63.6%) attributed the symptoms entirely to their dental practice, while 15 (45.5%) reported partial work-related causes. Although 31 (73.8 %) indicated awareness of ergonomic practices, only 25 (59.5%) applied them consistently in clinical settings. General dentists comprised 29 (69.0%) of the participants, with the 40-49 age group being most represented (17; 40.5%).

Conclusion

The findings reveal a concerning prevalence of MSDs among dentists practicing in PHCC settings, highlighting the urgent need for targeted preventive measures. Promoting ergonomic training, optimizing clinical schedules, and encouraging regular physical activity which may help mitigate the impact of MSDs. Furthermore, future policies should prioritize occupational health and ergonomic integration within primary dental care services.

## Introduction

Globally, musculoskeletal disorders (MSDs) contribute towards a significant burden of disease and pose serious health implications among a wide range of occupational groups [1,2]. MSDs are defined as a group of injuries and disorders that affect the musculoskeletal system. These disorders typically cause pain and discomfort that may worsen over time and interfere with daily activities. Examples of MSDs include lower back pain, neck strain, tendinitis, carpal tunnel syndrome, and shoulder impingement [1,2].

Evidence suggests that musculoskeletal disorders represent a significant global burden, affecting a wide range of occupational groups [3]. Health care professionals, particularly dentists, are among the most affected due to the physical demands of their work. Various studies have been conducted internationally to signify that dentists have higher risk of suffering from MSDs than the general population due to static and prolonged work time. In fact, the oral cavity represents a very small, confined working area, challenging dental practitioners to gain clear and sufficient access. In addition, various dental treatments require numerous physical strains (awkward positions, prolonged static posture, repetitive motions, and vibrations), which contribute to the development and exacerbation of MSDs in dental professionals [4]. Moreover, these disorders can considerably reduce productivity, quality of care and possibly lead to early retirement as well as affect mental wellbeing [5].

In Qatar, despite the growing emphasis on occupational well-being, research on MSDs among dental practitioners working in primary healthcare settings remains limited. Therefore, the aim of this study is to investigate the prevalence of musculoskeletal disorders among dentists working in the Primary Health Care Corporation (PHCC) in Qatar and to explore the associated occupational and ergonomic contributing factors.

## Materials and methods

Study design and setting

This study employed a descriptive, cross-sectional design to assess the prevalence of MSDs among dentists working in the PHCC in Qatar. Data were collected using a structured, self-administered online questionnaire developed in English and disseminated via official PHCC email channels using Microsoft Forms (Redmond, WA, USA). The study was reported in accordance with the Strengthening the Reporting of Observational Studies in Epidemiology (STROBE) checklist for cross-sectional studies [6].

Study participants

All licensed dentists actively practicing within PHCC at the time of the study, including general dentists, specialists, and consultants, were eligible to participate. Inclusion criteria required participants to be employed in a clinical role, possess an official PHCC email address, and provide informed consent by completing the online questionnaire. Dentists who were on extended leave (such as medical or maternity), not engaged in clinical duties (e.g., administrative-only roles), or who did not respond to the survey were excluded from the study. The total target population comprised 216 eligible dentists across PHCC health centers in Qatar.

Sample size and sampling technique

A census sampling approach was adopted. As the objective was to assess the entire population of PHCC dentists, no sample size calculation or random sampling was required. All eligible dentists were invited to participate between November 1 and November 30, 2024. A reminder email was sent two weeks after the initial invitation to maximize response rates.

Study tool and data collection

A structured, self-administered questionnaire was developed specifically for this study to assess musculoskeletal symptoms and related occupational factors among dentists working in primary healthcare settings. The content and structure of the questionnaire were scientifically inspired by the thematic domains of two widely recognized instruments: the Nordic Musculoskeletal Questionnaire (NMQ) and the Cornell Musculoskeletal Discomfort Questionnaire (CMDQ) [7,8].

It is important to clarify that no items, questions, or wording were directly reproduced from either instrument. The final questionnaire was custom-designed by the research team, tailored to the clinical context of dental practice in Qatar, and aligned with academic fair-use practices for non-commercial, original research. The tool comprised predominantly closed-ended questions and was organized into six key areas: it began with demographic data, followed by questions on musculoskeletal symptoms experienced over the past 12 months, including affected body regions and symptom duration. The third section focused on intervention strategies such as medications, physical therapy, or ergonomic tools. The fourth section explored the impact of symptoms on clinical performance, including sick leave or job role modifications. The fifth area assessed ergonomic awareness and the extent to which ergonomic practices were applied in daily work. Finally, the sixth section included an open-ended question inviting participants to share suggestions for improving workplace ergonomics and musculoskeletal health. The survey was administered in English via Microsoft Forms and distributed through official PHCC email channels.

Before starting the questionnaire, participants were presented with an introductory page explaining the study purpose, expected time to complete the survey (approximately 10 minutes), and emphasizing the value of honest responses. This page included a clear statement of voluntary participation, anonymity, confidentiality, and the right to withdraw at any time without consequences. Participants were informed that completing the questionnaire would be considered as providing implied informed consent. Contact details for the principal investigator were provided for any questions or clarifications.

As this study employed a self-administered, anonymous online questionnaire completed directly by the participating dentists without in-person interviews or clinical examinations, examiner calibration or inter-examiner reliability assessments were not applicable. Standardization of data collection was ensured through the use of a single, uniform, electronic survey instrument distributed via official PHCC email channels, with all participants receiving identical instructions and questions.

The questionnaire was custom-designed by the research team, composed of experienced dental clinicians and a clinical researcher specializing in occupational health. To ensure face validity and clarity, the draft questionnaire was pilot tested on a convenience sample of three PHCC dentists. Pilot participants were asked to comment on question clarity, language, comprehensiveness, and ease of completion. Feedback revealed minor issues, such as rewording ambiguous terms and adjusting the sequence of questions for logical flow; these revisions were incorporated into the final version. Because the tool consisted mainly of straightforward, closed-ended items focused on self-reported symptoms and ergonomic practices, and considering the minimal risk nature of the study, no formal psychometric reliability testing was performed. No formal psychometric validation, such as reliability or internal consistency testing (e.g., Cronbach’s alpha), was performed, as the questionnaire was designed for descriptive purposes to capture prevalence and distribution rather than to develop a measurement scale. No scoring system or composite indices were used; each categorical response was analyzed individually, and frequencies and percentages were reported separately.

The questionnaire was designed primarily with closed-ended categorical questions that did not require numerical scoring. Each response option was analyzed as a standalone categorical variable, reported as frequencies and percentages. For example, the presence or absence of musculoskeletal symptoms was coded as binary (yes/no), body regions with symptoms were recorded as multiple selections, and frequency of symptoms was categorized into ordinal ranges (e.g., occasionally, sometimes, frequently). No composite scores or rating scales were calculated, as the study focused on the prevalence and distribution of self-reported symptoms rather than quantitative severity indices.

Ethical considerations

Ethical approval for this study was obtained from the Clinical Research Department of the Primary Health Care Corporation (PHCC), Qatar, under ethical clearance number BUHOOTH-D-24-00064. Participation was entirely voluntary. Informed consent was obtained electronically: on the first page of the online questionnaire, participants were provided with clear information about the study objectives, estimated time required (approximately 10 minutes), the voluntary nature of participation, data confidentiality, and their right to skip questions or withdraw at any time without consequences. Specific statements included: “Your responses are anonymous and confidential,” “Participation is voluntary, and you may withdraw at any time,” and “The data collected will be used solely for research purposes.” Completion of the online questionnaire was considered as providing implied informed consent. Contact information for the principal investigator was provided in the questionnaire invitation to allow participants to ask questions or request clarifications. All responses were anonymized and stored securely in a password-protected database, adhering to the ethical principles outlined in the Declaration of Helsinki and institutional guidelines for human subject research.

Data analysis

Data collected through Microsoft Forms were exported to Excel and subsequently analyzed using IBM SPSS Statistics version 28.0 (IBM Corp., Armonk, NY, USA). Descriptive statistics were used to summarize participants’ demographic and clinical characteristics. Categorical variables were presented as frequencies and percentages, while continuous variables were expressed as means and standard deviations. The Shapiro-Wilk test was used to assess the normality of continuous variables (height and weight), and both variables were found to be normally distributed (p > 0.05), supporting the use of parametric descriptive statistics. To explore associations between independent variables and the presence of MSDs, univariate logistic regression analyses were performed. Variables demonstrating a p-value <0.10 in univariate analysis were included in the multivariate logistic regression model to adjust for potential confounders. Age and gender were included in the multivariate model a priori, given their established relevance in occupational health research. Prevalence odds ratios (PORs) with corresponding 95% confidence intervals (CIs) were calculated to assess the strength and direction of associations. Model calibration was assessed using the Hosmer-Lemeshow goodness-of-fit test. A two-sided p-value <0.05 was considered statistically significant for all analyses. As all questions in the online questionnaire were marked as required, no missing data were encountered; therefore, no imputation or exclusion techniques were necessary during analysis.

## Results

Response rate and participant characteristics

A total of 42 dentists working in PHCC completed the questionnaire, yielding a response rate of 19.4% of the total PHCC dental workforce (n = 216). Among participants, 23 (54.8%) were male and 19 (45.2%) were female. Most respondents were aged between 40 and 49 years (17; 40.5%), followed by 30-39 years (11; 26.2%), 50-59 years (10; 23.8%), and over 60 years (4; 9.5%). Most participants were general dentists (29; 69.0%), with specialists (10; 23.8%) and consultants (3; 7.1%) comprising the remainder. All participants were non-Qatari nationals. Regarding professional experience, 35 (83.3%) had over 10 years of clinical experience, while five (11.9%) had between five and 10 years, and two (4.8%) had two to five years. Most dentists were right-handed (37; 88.1%), with a mean height of 170.9 ± 10.1 cm and a mean weight of 82.7 ± 15.5 kg (Table 1).

**Table 1:** Participant characteristics of dentists working in primary healthcare settings Demographic and professional characteristics of respondents, including age group, gender, job title, years of experience, handedness, and anthropometric measurements. Percentages are based on the total sample (N = 42).

Characteristic	Category	n	%
Gender	Male	23	54.8
	Female	19	45.2
Age Group	40-49	17	40.5
	30-39	11	26.2
	50-59	10	23.8
	Over 60	4	9.5
Job Title	General Dentist	29	69
	Specialist	10	23.8
	Consultant	3	7.1
Nationality	Non-Qatari	42	100
	Qatari	0	0
Years of Experience	2 to 5 years	2	4.8
	5 to 10 years	5	11.9
	Over 10 years	35	83.3
Dominant Hand	Right-Handed	37	88.1
	Left-Handed	4	9.5
	Both	1	2.4
Height (cm)	Mean (SD)		170.9 ± 10.1
Weight (kg)	Mean (SD)		82.7 ± 15.5

Prevalence and distribution of musculoskeletal complaints

Musculoskeletal complaints within the previous 12 months were reported by 78.6% of respondents (n = 33). The distribution of symptoms by body region reflects the percentage of total selections made across all body areas rather than the number of individuals affected. The most frequently reported body regions were the neck (27; 22.3%), shoulders (26; 21.4%), and lumbar back (24; 19.8%), followed by wrist/hands (11; 9.1%), knees (10; 8.3%), thoracic back (9; 7.4%), elbows (5; 4.1%), ankles/feet (5; 4.1%), and hips/thighs (4; 3.3%). These percentages are based on a total of 121 regional symptom selections, illustrating how frequently each body part was cited in relation to overall complaints (Table 2). Overall, a substantial proportion of dentists reported multiple affected regions, particularly involving the neck, shoulders, and lower back, reflecting the high physical demands of dental practice.

**Table 2:** Prevalence of musculoskeletal symptoms by body region among Primary Health Care Corporation dentists Distribution of self-reported musculoskeletal complaints across various body regions. Percentages are based on the total number of regional symptom selections (N = 121).

Body Region	Affected Dentists (n)	Percentage (%)
Neck	27	22.3
Shoulder	26	21.4
Lower Back	24	19.8
Wrist/hand	11	9
Knees	10	8.2
Thoracic Back	9	7.4
Hips/Thighs	4	3.3
Elbows	5	4.1
Ankles/Feet	5	4.1

Consequences of musculoskeletal symptoms

Among the 33 participants who reported musculoskeletal complaints, 15 (45.5%) indicated that they had taken sick leave due to their symptoms. Twenty-one (21; 63.6%) respondents attributed their symptoms entirely to their occupational duties as dentists, while 15 (45.5%) considered their discomfort to be partly work-related, and six (18.2%) believed it was unrelated to their job. Additionally, 17 (51.5%) participants reported a reduction in work activity at home or in the clinic, and 10 (30.3%) had to change their job roles or duties, even temporarily, due to musculoskeletal issues. More than half of the respondents, 23 (69.7%), sought professional consultation with doctors, physiotherapists, or chiropractors, and nine (27.3%) were hospitalized as a result of their discomfort (Table 3).

**Table 3:** Consequence of musculoskeletal symptoms among dentists reporting musculoskeletal disorders (MSDs) Impact of musculoskeletal symptoms on work and clinical functioning among affected participants. Percentages are based on those who reported MSDs (N = 33). Note: Percentages may exceed 100% because participants were allowed to select more than one attribution category.

Consequence	Number of Responses (n)	Percentage (%)
Sick leave due to MSDs	15	45.5
Attribution solely to occupational causes	21	63.6
Attribution to mixed causes	15	45.5
Attribution to non-occupational causes	6	18.2
Reduction in clinical activity	17	51.5
Job or duty change due to symptoms	10	30.3
Consultation with healthcare professional	23	69.7
Hospitalisation due to MSDs	9	27.3
Took medication for ache or discomfort	28	84.8

In terms of symptom frequency over the past year, 12 (36.4%) participants reported experiencing pain frequently (more than 30 days), 11 (33.3) occasionally (1-7 days), six (18.2%) sometimes (8-30 days), and six (18.2%) constantly (every day), while seven (21.2%) indicated they never experienced discomfort (Table 4).

**Table 4:** Frequency of symptoms (duration over the past year) Reported frequency of musculoskeletal pain episodes within the last 12 months among participants with musculoskeletal disorders. Percentages are based on N = 33.

Frequency of Pain (past year)	n	%
Never	7	21.2%
Occasionally (1–7 days)	11	33.3%
Sometimes (8–30 days)	6	18.2%
Frequently (>30 days)	12	36.4%
Constantly (every day)	6	18.2%

Regarding medication use, 28 (84.8%) participants reported taking medications to manage their symptoms, whereas five (15.2%) did not (Table 5).

**Table 5:** Use of medication to manage musculoskeletal symptoms Self-reported use of medication to manage musculoskeletal discomfort among respondents with musculoskeletal disorders (MSDs). Percentages are based on N = 33.

Took Medication for MSDs	n	%
Yes	28	84.8%
No	5	15.2%

To prevent or alleviate musculoskeletal symptoms, participants reported various techniques: stretching exercises were used by 35 respondents (35; 47.3% of all selections), followed by hot baths (20 selections, 27.0%), back patches (14 selections, 18.9%), and cupping therapy (5 selections, 6.8%) (Table 6). Despite the significant impact of musculoskeletal disorders, these findings suggest that preventive strategies and ergonomic interventions remain underutilized among primary healthcare dentists, underscoring the need for institutional support and improved awareness. 

**Table 6:** Technique used by dentists to relieve musculoskeletal symptoms Multiple-response data showing the techniques used by participants to alleviate musculoskeletal discomfort. Percentages are based on the total number of technique selections (N = 74).

Relief Technique	n (Selections)	% of Total Selections (n = 74)
Stretching Exercises	35	47.3%
Hot Baths	20	27.0%
Back Patches	14	18.9%
Cupping Therapy	5	6.8%

Ergonomic knowledge and practices

The majority of dentists (31 out of 42; 73.8%) reported having knowledge of dental ergonomics, indicating a generally high level of awareness within the PHCC dental workforce. However, only 25 respondents (59.5%) reported consistently applying ergonomic principles during their daily clinical activities. Among those who indicated awareness, 80.6% (25 out of 31) actively implemented ergonomic strategies, suggesting a strong but not universal translation of knowledge into practice. Conversely, 11 participants (26.2%) lacked ergonomic knowledge, and 17 (40.5%) reported that they did not apply ergonomic practices, even if they were aware of them. This highlights a substantial knowledge-practice gap, which may be influenced by factors such as lack of ergonomic equipment, clinical time pressures, or insufficient institutional emphasis on preventive ergonomics.

This gap is particularly relevant given the high burden of MSDs reported in this study. While awareness is a critical first step, consistent application of ergonomic practices is essential to reduce the risk and impact of MSDs. These findings suggest an opportunity for targeted educational interventions, reinforcement through policy, and improved access to ergonomic tools to promote safer clinical environments (Table 7).

**Table 7:** Knowledge and application of ergonomics practices among Primary Health Care Corporation dentists Self-reported awareness and daily application of ergonomic practices among all surveyed participants. Percentages are based on the total sample (N = 42).

Category	Yes (n)	No (n)	Yes (%)	No (%)
Knowledge of Ergonomics	31	11	73.8	26.2
Application of Ergonomics	25	17	59.5	40.5

Association between risk factors and musculoskeletal disorders

To explore potential contributors to MSDs, univariate logistic regression analyses were performed. These analyses examined the relationship between individual characteristics, ergonomic behavior, and the presence of MSDs among dentists. The application of ergonomic principles demonstrated a protective trend against MSDs (OR = 0.23; 95% CI: 0.03-1.48; P = 0.118), although this association did not reach statistical significance. Other demographic and professional variables, including gender, age group, job title, years of experience, and ergonomic knowledge, also showed no statistically significant associations with MSD occurrence in univariate models. However, the direction of some trends such as increased MSD risk with advancing age and among female dentists was consistent with findings from previous studies.

Multivariate logistic regression was subsequently conducted, adjusting for age and gender regardless of their univariate significance. In this model, the application of ergonomic principles continued to show a non-significant protective effect (OR = 0.27; 95% CI: 0.04-1.82; P = 0.176). Dentists aged 40 years and older had higher odds of reporting MSDs (OR = 2.82; 95% CI: 0.43-18.31; P = 0.277), while female dentists exhibited increased odds compared to their male counterparts (OR = 1.84; 95% CI: 0.35-9.62; P = 0.470). None of these associations reached statistical significance, likely due to the limited sample size (Table 8).

**Table 8:** Multivariate logistic regression analysis of risk factors associated with musculoskeletal disorder among Primary Health Care Corporation dentists Results of logistic regression analysis evaluating independent associations between selected predictors and MSD prevalence. Odds ratios are adjusted for age and gender. Statistical significance was defined as p < 0.05.

Risk Factor	Odds Ratio (OR)	95 % Confidence Interval (CI)	P-Value
Age group (>40 years vs <40 years)	2.82	0.43-18.31	0.277
Gender (Female vs Male)	1.84	0.35-9.62	0.47
Application of ergonomics	0.27	0.04-1.82	0.176

Additionally, clinical workload was examined as a contextual factor. The majority of respondents treated six to 10 patients per day (23; 54.8%), followed by 11 to 15 patients per day (17; 40.5%), while only two participants reported treating fewer than six or more than 15 patients daily. Although not formally analyzed due to power limitations, a higher patient volume may be associated with increased physical strain, reduced microbreak opportunities, and sustained awkward postures factors previously identified as contributors to MSD risk in dental professionals. Future studies should consider including patient load as a key variable in multivariate models exploring ergonomic risk.

Suggestions and qualitative insights

Open-ended responses provided valuable perspectives on strategies to prevent and manage MSDs in the dental workplace. While the study was primarily quantitative, participants’ comments were thematically analyzed to extract common areas of concern and suggested improvements. Thematic categorization revealed four main areas: ergonomic awareness, workplace design, workload and scheduling, and health and wellness support.

Participants highlighted the need for enhanced ergonomic training through workshops and integration into continuing professional development (CPD). Several comments addressed inadequate access to ergonomic tools and poor workstation design, emphasizing the need for better equipment and adjustments. Workload-related stressors were frequently mentioned, with calls for reducing patient volume and incorporating regular microbreaks into daily schedules. Health promotion initiatives such as stretching, exercise, and access to physiotherapy were also recommended to support physical resilience.

These insights, summarized in Table 9, provide practical direction for developing targeted interventions to reduce the burden of MSDs among dental professionals.

**Table 9:** Themes and subthemes from open-ended participants responses on musculoskeletal disorder (MSD) prevention Summary of key themes and illustrative quotes from participant suggestions on MSD prevention. Themes include ergonomic awareness, workplace equipment, workload, and wellness support. CPD: continuing professional development

Theme	Sub-theme	Illustrative Quote						
Ergonomic Awareness & Training	Need for ergonomic workshops	We need regular ergonomic training to avoid wrong posture habits.						
	Integration into CPD	CPD courses should include ergonomics and posture correction.						
Workplace Design & Equipment	Inadequate ergonomic tools	Loupes and adjustable chairs should be made available in all clinics.						
	Workstation adjustments	Recognizing workstations can reduce strain on the neck and back.						
Workload and Scheduling	High patient load	Reducing number of patients per day will help to avoid burnout.						
	Lack of micro-breaks	Short breaks between patients would relieve physical stress.						
Health and Wellness Support	Physical fitness and stretching	Daily stretching should be part of our routine.						
	Access to physiotherapy	Providing in-house physiotherapy sessions would be beneficial.						

## Discussion

This cross-sectional study identified a high prevalence of MSDs, affecting 33 of 42 dentists (78.6%) practicing in primary healthcare settings across Qatar. This finding aligns closely with global estimates, such as the pooled prevalence of 78.4% reported in a 2022 meta-analysis by Chenna et al., underscoring the substantial and widespread occupational health burden posed by MSDs within the dental profession [9]. Similarly elevated prevalence rates have been documented in European studies, including national surveys reporting MSD prevalence of 78.8% in Portugal and 81.9% in Italy [10]. In Germany, dental professionals exhibited particularly high rates, with seven-day, 12-month, and lifetime prevalences of 86.9%, 97.5%, and 98.5%, respectively, with the neck identified as the most frequently affected region [11]. Kierklo et al. reported a prevalence of 79% among Polish dentists [12], while Finsen et al. documented an 85% prevalence among dental practitioners in Scandinavian countries [13]. Collectively, these findings reinforce that MSDs represent a pervasive and significant global occupational hazard within the dental profession.

In our cohort, the neck (27 of 121 regional selections; 22.3%), shoulders (26; 21.4%), and lower back (24; 19.8%) emerged as the most commonly affected anatomical regions over the past 12 months, a distribution consistent with global patterns. A recent Indonesian study reported an overall prevalence of 96% for work-related MSDs among dental professionals, with the back (68.2%), waist (66.9%), upper neck (60.9%), and lower neck (59.6%) most frequently affected, further highlighting the global burden [14]. Likewise, a meta-analysis by Lietz et al. found the neck (58.5%), lower back (56.4%), and shoulders (43.1%) as the most commonly affected regions among dental practitioners in Western countries [15,16]. Nordin et al. reported a 12-month prevalence of 64.5% for neck pain and 61% for lower back pain among Swedish dentists [17], while Leggat and Smith observed similar rates of neck (68%) and lumbar (54%) discomfort among dentists in Australia [18]. These consistently elevated rates of musculoskeletal symptoms, particularly involving the neck, shoulders, and lower back, emphasize the urgent need for targeted ergonomic interventions in dental practice. These prevalence patterns provide important context for understanding the occupational consequences reported in our study.

In our study, 15 of 33 symptomatic dentists (45.5%) reported taking sick leave due to musculoskeletal symptoms, while 17 (51.5%) indicated a reduction in clinical activity, underscoring the significant occupational impact of MSDs on work capacity and professional performance. Valachi and Valachi demonstrated that prolonged static postures increase spinal disc loading and contribute to muscular ischemia, which can lead to chronic neck and lumbar pain adversely affecting practitioner well-being and patient care [19]. In India, Muralidharan et al. found that 78% of dentists experienced work-related MSDs, with neck pain (52%) and lower back pain (40%) being the most prevalent symptoms [20]. Szymańska et al. observed that 48% of Polish dentists reported low back pain, with awkward working positions identified as primary contributors [21]. Chiu et al. confirmed that MSDs significantly impair dentists’ work ability, leading to decreased productivity and intentions to retire prematurely [22]. Moreover, Smith et al. [23] highlighted that early onset of musculoskeletal symptoms can significantly impair dentists’ work ability, potentially leading to reduced productivity or premature departure from clinical practice. This underscores the importance of early preventive strategies to protect long-term occupational health. This evidence demonstrates that MSDs contribute to absenteeism, reduced work capacity, and early workforce exit, highlighting the need for early ergonomic interventions, micro-break scheduling, and institutional support.

Our findings revealed that 12 of 33 symptomatic dentists (36.4%) reported frequent pain episodes lasting over 30 days annually, and six (18.2%) experienced constant daily pain, underscoring the chronic nature of MSDs among dental professionals. Chronic pain has been linked to reduced work ability, increased stress, and a higher risk of premature retirement, emphasizing the urgency of timely interventions. Notably, 28 of 33 participants (84.8%) relied on medications to manage discomfort, reflecting a heavy dependence on pharmacologic treatments and suggesting limited use of comprehensive non-pharmacologic strategies such as physiotherapy or ergonomic retraining.

Although 35 participants (47.3% of technique selections) reported practicing stretching exercises, adoption of other conservative methods, including postural correction or micro-breaks, was limited. This low engagement likely reflects systemic barriers, such as time constraints and inadequate ergonomic training. Moreover, while 31 of 42 dentists (73.8%) reported awareness of ergonomic principles, only 25 (59.5%) applied them consistently in daily practice, revealing a critical knowledge-practice gap that must be addressed through enhanced institutional support, improved access to ergonomic equipment, and continuous professional development.

Regarding workload, most respondents treated between six and 15 patients daily, a level likely to increase the risk of cumulative musculoskeletal strain due to fewer opportunities for posture variation or rest. High daily patient volumes have been consistently associated with increased MSD risk, underscoring the need for workload optimization strategies to mitigate ergonomic hazards.

Attribution patterns in our study were also revealing: 21 of 33 symptomatic dentists (63.6%) identified dental work as the sole cause of their MSDs, while 15 (45.5%) attributed symptoms partially to occupational factors, reinforcing the strong perceived link between clinical practice and musculoskeletal issues. Additionally, qualitative insights from participants highlighted the need for ergonomic workshops, improved ergonomic tools, adjustments in workload, and workplace wellness programs recommendations that align with international best practices and should inform institutional policies aimed at reducing MSD risk. These practical insights are critical for informing targeted interventions and organizational policies.

Overall, our findings confirm that MSDs remain a widespread occupational concern consistent with global literature. They highlight the urgent need for comprehensive strategies including ergonomic training, improved clinic design, workload adjustments, and wellness initiatives to reduce the burden of MSDs and enhance the well-being and career longevity of dental professionals.

Strengths and limitations

This study presents several strengths. It focused on primary healthcare dentists, a population often underrepresented in occupational health research. The use of a standardized self-administered questionnaire allowed for consistent data collection across participants, minimizing interviewer bias. Furthermore, the study explored both physical and ergonomic risk factors, supplemented by qualitative insights that provided a broader understanding of preventive needs within this setting. However, some limitations should be acknowledged. The relatively small sample size (n = 42) reduced the statistical power of the study, potentially limiting the ability to detect significant associations. The cross-sectional design precludes any inference of causality between risk factors and musculoskeletal disorders. Additionally, the reliance on self-reported data introduces the possibility of recall and reporting bias. Finally, since all participants were non-Qatari dentists, the generalizability of findings to broader or nationally representative dental populations may be limited [23,24].

Clinical implications and recommendations

Based on the findings of this study, several policy-level and institutional recommendations are proposed to reduce the burden of MSDs among dentists working in primary healthcare settings. They include the following suggestions.

Continuous revision and improvement of regular ergonomic training programs targeting dental practitioners who are at high risk of work-related MSD is suggested. These sessions can be feasibly integrated into existing CPD programs already implemented within PHCC and should focus on reinforcing proper posture, safe instrument handling, and physical load management strategies during dental procedures [25].

Access to ergonomic equipment can be further improved and upscaled across all PHCC dental clinics. This includes ergonomic dental chairs, operator stools, magnification aids, and proper lighting systems. Ensuring the availability of such equipment can facilitate better postural alignment and reduce repetitive strain injuries [26].

Organizational modifications aimed at reducing workload-related risk factors should be prioritized. These include promoting micro-breaks between patient appointments, maintaining manageable daily patient volumes, and optimizing patient scheduling. Encouraging variation in clinical tasks can also help mitigate the impact of static postures and repetitive movements [27].

Preventive health and wellness initiatives can be further upscaled. The workplace wellness programs such as access to physiotherapy, daily stretching routines, and stress management workshops can be re-evaluated, incorporating feedback by health care staff. These strategies can help maintain musculoskeletal health and reduce absenteeism due to pain or injury [28]. Additionally, untreated or recurrent MSDs may lead to chronic pain, disability, and premature retirement, which could exacerbate workforce shortages and impact healthcare system sustainability. Recognizing these long-term consequences underscores the urgency of implementing preventive measures and supporting dentist well-being. Finally, further research is encouraged to monitor MSD trends and evaluate the effectiveness of implemented interventions.

Future studies with larger, more diverse populations and longitudinal designs are essential to establish causality, identify systemic barriers to ergonomic practice, and assess the long-term outcomes of targeted ergonomic and wellness strategies [29].

## Conclusions

This study revealed a high prevalence of MSDs among dentists working in primary healthcare settings in Qatar, with the neck, shoulders, and lower back being the most affected regions. While most participants reported awareness of ergonomic practices, a substantial proportion did not consistently apply them in daily clinical work. Although the associations between MSDs and demographic variables such as age and gender did not reach statistical significance, trends indicated a potential increased risk among older and female dentists. These findings highlight the importance of integrating ergonomic training, promoting workload adjustments, and supporting preventive wellness initiatives within clinical settings. Urgent institutional ergonomic reform through structured training, workplace redesign, and wellness integration is essential to protect dental practitioners from preventable burnout and long-term disability.

## Appendices

**Table 10:** Self-Developed Questionnaire The following table presents the full set of questions used in the self-developed questionnaire to assess musculoskeletal disorders among Primary Health Care Corporation dentists.

Section	Q. No.	Question	Response Options
A. Demographic Data	1	What is your job title?	General Dentist / Specialist / Consultant
A. Demographic Data	2	What is your age?	20–29 / 30–39 / 40–49 / 50+
A. Demographic Data	3	What is your gender?	Male / Female
A. Demographic Data	4	What is your nationality?	Qatari / Non-Qatari
A. Demographic Data	5	What is your height? (cm)	Open field
A. Demographic Data	6	What is your weight? (kg)	Open field
A. Demographic Data	7	How many years of experience do you have in dentistry?	<1 / 1–2 / 2–5 / 5–10 / >10
A. Demographic Data	8	Are you right-handed or left-handed?	Right / Left / Both
B. Musculoskeletal Symptoms	9	Have you experienced any ache, pain, or discomfort in any part of your body during the last 12 months?	Yes / No
B. Musculoskeletal Symptoms	10	In which part of your body have you experienced ache, pain, or discomfort?	Neck / Shoulder / Thoracic back / Elbow / Lumbar back / Hand/Wrist / Hip/Thigh / Knee / Foot/Ankle
B. Musculoskeletal Symptoms	11	Have you ever been hospitalized because of this ache, pain, or discomfort?	Yes / No
B. Musculoskeletal Symptoms	12	How frequently have you experienced pain or discomfort over the past year?	Never / Occasionally (1–7 days) / Sometimes (8–30 days) / Frequently (>30 days) / Constantly (every day)
C. Interventions strategy	13	Have you taken medication for ache, pain, or discomfort during the last 12 months?	Yes / No
C. Interventions strategy	14	What techniques have you used to prevent musculoskeletal disorders?	Hot bath / Cupping therapy / Stretching exercises / Back patches (Select all that apply)
C. Interventions strategy	15	Have you consulted a doctor, physiotherapist, chiropractor, or other professionals for ache, pain, or discomfort in the last 12 months?	Yes / No
D. Impact on Professional Activity	16	Has ache, pain, or discomfort caused you to reduce your work activity during the last 12 months?	Yes / No
D. Impact on Professional Activity	17	Have you had to change jobs or duties because of ache, pain, or discomfort?	Yes / No
D. Workload and Ergonomic Practices	18	Have you taken sick leave due to ache, pain, or discomfort during the last 12 months?	Yes / No
D. Workload and Ergonomic Practices	19	Do you think your ache, pain, or discomfort is related to your current work as a dentist?	Solely related / Partly related / Not related
E. Workload and Ergonomic Practices	20	What is the average number of patients you treat per day?	1–5 / 6–10 / 11–15 / More than 15
E. Workload and Ergonomic Practices	21	Do you have knowledge of dental ergonomics?	Yes / No
E. Workload and Ergonomic Practices	22	Do you apply ergonomics in your dental practice?	Yes / No
F. Recommendations for Prevention	23	What do you think would help reduce your ache, pain, or discomfort?	Micro break time / Workstation adjustments / Training on posture and stress reduction / Increasing fitness level (Select all that apply)
F. Recommendations for Prevention	24	Do you have any other suggestions?	Open field

## References

[1] Liu S, Wang B, Fan S, Wang Y, Zhan Y, Ye D (2022). Global burden of musculoskeletal disorders and attributable factors in 204 countries and territories: a secondary analysis of the Global Burden of Disease 2019 study. BMJ Open.

[2] (2023). Global, regional, and national burden of other musculoskeletal disorders, 1990-2020, and projections to 2050: a systematic analysis of the Global Burden of Disease Study 2021. Lancet Rheumatol.

[3] Jacquier-Bret J, Gorce P (2023). Prevalence of body area work-related musculoskeletal disorders among healthcare professionals: a systematic review. Int J Environ Res Public Health.

[4] Soo SY, Ang WS, Chong CH, Tew IM, Yahya NA (2023). Occupational ergonomics and related musculoskeletal disorders among dentists: a systematic review. Work.

[5] Hayes MJ, Smith DR, Taylor JA (2013). Musculoskeletal disorders and symptom severity among Australian dental hygienists. BMC Res Notes.

[6] Ghaferi AA, Schwartz TA, Pawlik TM (2021). STROBE reporting guidelines for observational studies. JAMA Surg.

[7] Kuorinka I, Jonsson B, Kilbom A (1987). Standardised Nordic questionnaires for the analysis of musculoskeletal symptoms. Appl Ergon.

[8] Hedge A, Morimoto S, McCrobie D (1999). Effects of keyboard tray geometry on upper body posture and comfort. Ergonomics.

[9] Chenna D, Pentapati KC, Kumar M, Madi M, Siddiq H (2022). Prevalence of musculoskeletal disorders among dental healthcare providers: a systematic review and meta-analysis. F1000Res.

[10] Bracciale A, Manso MC, Bracciale F (2025). Prevalence of self-reported musculoskeletal disorders in dentists—a cross-sectional study in Portugal and Italy. Healthcare.

[11] Holzgreve F, Haas Y, Naser A (2021). Prevalence of musculoskeletal disorders in Germany—a comparison between dentists and dental assistants. Appl Sci.

[12] Kierklo A, Kobus A, Jaworska M, Botuliński B (2011). Work-related musculoskeletal disorders among dentists - a questionnaire survey. Ann Agric Environ Med.

[13] Finsen L, Christensen H, Bakke M (1998). Musculoskeletal disorders among dentists and variation in dental work. Appl Ergon.

[14] Kholinne E, Azalia X, Rahayu EP, Anestessia IJ, Agil N, Muchtar Muchtar (2025). The prevalence and risk factors of musculoskeletal disorders among Indonesian dental professionals. Front Rehabil Sci.

[15] Lietz J, Kozak A, Nienhaus A (2018). Prevalence and occupational risk factors of musculoskeletal diseases and pain among dental professionals in Western countries: a systematic literature review and meta-analysis. PLoS One.

[16] Rundcrantz BL, Johnsson B, Moritz U (1991). Pain and discomfort in the musculoskeletal system among dentists: a prospective study. Swed Dent J.

[17] Leggat PA, Smith DR (2006). Musculoskeletal disorders self-reported by dentists in Queensland, Australia. Aust Dent J.

[18] Valachi B, Valachi K (2003). Preventing musculoskeletal disorders in clinical dentistry: strategies to address the mechanisms leading to musculoskeletal disorders. J Am Dent Assoc.

[19] Muralidharan D, Fareed N, Shanthi M (2013). Musculoskeletal disorders among dental practitioners: does it affect practice?. Epidemiol Res Int.

[20] Szymańska J (2002). Disorders of the musculoskeletal system among dentists from the aspect of ergonomics and prophylaxis. Ann Agric Environ Med.

[21] Xiang J, Hansen A, Pisaniello D, Dear K, Bi P (2018). Correlates of occupational heat-induced illness costs: case study of South Australia 2000 to 2014. J Occup Environ Med.

[22] Feng B, Liang Q, Wang Y, Andersen LL, Szeto G (2014). Prevalence of work-related musculoskeletal symptoms of the neck and upper extremity among dentists in China. BMJ Open.

[23] Alghadir A, Zafar H, Iqbal ZA (2015). Work-related musculoskeletal disorders among dental professionals in Saudi Arabia. J Phys Ther Sci.

[24] Garbin AJ, Garbin CA, Diniz DG, Yarid SD (2011). Dental students' knowledge of ergonomic postural requirements and their application during clinical care. Eur J Dent Educ.

[25] Mulimani P, Hoe VC, Hayes MJ, Idiculla JJ, Abas AB, Karanth L (2018). Ergonomic interventions for preventing musculoskeletal disorders in dental care practitioners. Cochrane Database Syst Rev.

[26] Hussein A, Mando M, Radisauskas R (2022). Work-related musculoskeletal disorders among dentists in the United Arab Emirates: a cross-sectional study. Medicina.

[27] Enone LL, Oyapero A, Ijarogbe O, Adeyemi TE, Ojikutu RO (2021). Ergonomic risks and prevalence of musculoskeletal disorders among dental surgeons in Nigeria: a descriptive survey. J Int Oral Health.

[28] Lietz J, Ulusoy N, Nienhaus A (2020). Prevention of musculoskeletal diseases and pain among dental professionals through ergonomic interventions: a systematic literature review. Int J Environ Res Public Health.

[29] Black RC (2006). The future of dentistry: challenges and opportunities, part 1. Tex Dent J.

